# Racial/Ethnic Disparities in Antimicrobial Drug Use, United States, 2014–2015

**DOI:** 10.3201/eid2411.180762

**Published:** 2018-11

**Authors:** Scott W. Olesen, Yonatan H. Grad

**Affiliations:** Harvard T.H. Chan School of Public Health, Boston, Massachusetts, USA (S.W. Olesen, Y.H. Grad);; Brigham and Women’s Hospital, Boston (Y.H. Grad)

**Keywords:** antibacterial agents, ethnic groups, population groups, healthcare disparities, antimicrobial drugs, national survey, racial disparities, antimicrobial drug stewardship, pharmacies, antimicrobial resistance, United States, USA, race/ethnicity

## Abstract

Using a US nationwide survey, we measured disparities in antimicrobial drug acquisition by race/ethnicity for 2014–2015. White persons reported twice as many antimicrobial drug prescription fills per capita as persons of other race/ethnicities. Characterizing antimicrobial drug use by demographic might improve antimicrobial drug stewardship and help address antimicrobial drug resistance.

Antimicrobial drug use varies by sex, age, and geography (*1*), and antimicrobial drug prescribing practice for specific medical conditions and age cohorts varies by patients’ race/ethnicity (*2*–*4*). Many studies on the role of patient race/ethnicity in antimicrobial drug prescribing practice focus on acute respiratory illnesses because antimicrobial drugs are often inappropriately prescribed for these conditions. The subjective diagnostic criteria for respiratory illnesses might result in race/ethnicity influencing prescribing practice more for these illnesses than for other illnesses (*4*). Despite our increasing knowledge of the role of patient race/ethnicity in drug prescribing practice for specific conditions, how or whether these specific effects translate into overall antimicrobial drug use by race/ethnicity remains unclear. In this report, we address this gap in knowledge by describing the extent of racial/ethnic disparities in overall antimicrobial drug prescription fill rates in the United States.

We used the nationwide Medical Expenditure Panel Survey (MEPS) to acquire data about race/ethnicity and outpatient antimicrobial drug use for 2014–2015, the latest years with data available. MEPS contains data on members of a nationally representative sample of households (*5*,*6*). A person’s race/ethnicity is reported by the respondent and imputed in <0.1% of cases. Information about race and Hispanic ethnicity are collected in separate questions. Data regarding prescriptions filled at outpatient pharmacies are collected from the respondent and, if the respondent approves, verified with the filling pharmacy. Data are afterward cross-referenced and cleaned by survey preparers (*5*).

We used 2 exposure variables. The first was a categorical race/ethnicity variable with 5 values: Hispanic, non-Hispanic white only, non-Hispanic black only, non-Hispanic Asian only, and non-Hispanic other or multiple race. The second exposure variable was dichotomous and indicated whether white was the race or 1 of the races of the respondent. This exposure variable included all persons from the non-Hispanic white category, some from the Hispanic category, and some from the other or multiple race category. The main outcome was reported outpatient antimicrobial drug prescription fills per 1,000 persons per year stratified by major antimicrobial drug class (penicillins, macrolides, quinolones, sulfonamides, other). The complex survey design was accounted for when computing rates and CIs with survey package version 3.32 in R version 3.4.1 (Technical Appendix).

The reported annual outpatient prescription fill rate for all antimicrobial drugs was 373 (95% CI 358–388) fills/1,000 persons. This rate varied by race/ethnicity; non-Hispanic whites reported the highest rate, followed by persons of other or multiple race/ethnicity, Hispanics, non-Hispanic blacks, and non-Hispanic Asians (Figure, panel A). White persons reported 2.0 (95% CI 1.9–2.2)–fold more fills per capita than nonwhite persons (Figure, panel B). The reported fill rate disparity was similar for macrolides (2.0 [95% CI 1.8–2.4]–fold higher), sulfonamides (2.2 [95% CI 1.8–2.7]–fold higher), and quinolones (2.3 [95% CI 1.9–2.8]–fold higher) but smaller for penicillins (64% [95% CI 48%–82%] higher).

**Figure:**
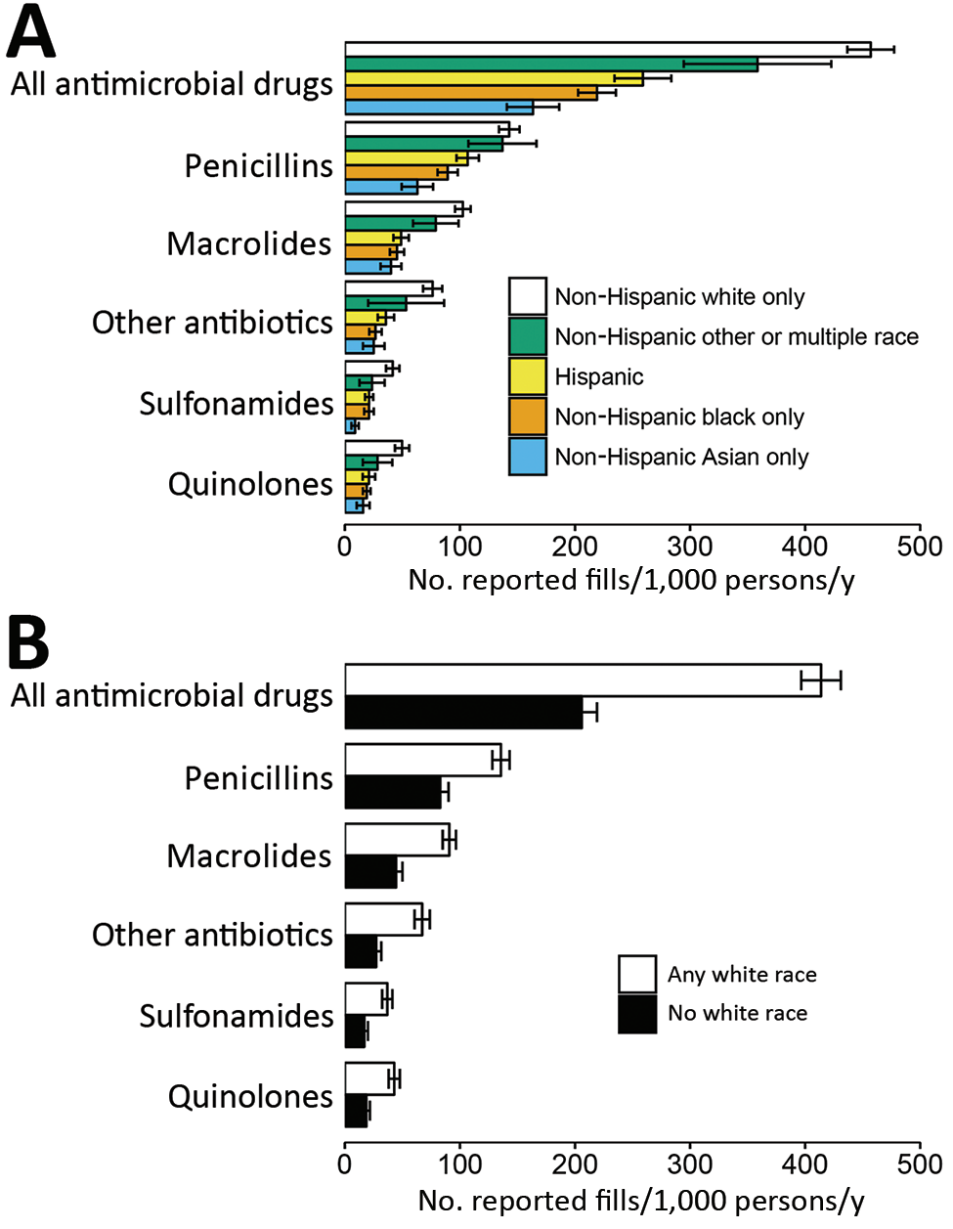
Annual antimicrobial drug use reported by Medical Expenditure Panel Survey respondents, by race/ethnicity, United States, 2014–2015. Error bars indicate 95% CIs. A) Drug use by race/ethnicity category. B) Drug use among persons who reported white as their race or 1 of their races and among those who did not.

We found a large disparity in antimicrobial drug fill rates by race/ethnicity: white persons reported making twice as many antimicrobial drug prescription fills as persons who were not white. Disparities were apparent for each major antimicrobial drug class, rather than different drug classes being used more predominantly by persons of particular race/ethnicities.

This study is subject to several limitations. First, the survey is not a perfect measure of antimicrobial drug use. Survey respondents report medications they remember filling, subjecting results to systematic differences in respondents’ abilities to recall medications (*6*). Survey preparers then obtain information about those medications from the pharmacies the respondents visited, which themselves might have systematic differences in completeness of records (*5*). Also, respondents might not have complete information about other household members’ antimicrobial drug use or might choose not to disclose all antimicrobial drug use. Second, nonprescription antimicrobial drug use might be higher among minority groups (*7*), perhaps mitigating the observed disparity. Last, the survey measures reported antimicrobial drug fills and not actual use (*8*); the fill rates we report are substantially lower than those measured by others using sales data (*1*) or other national surveys (*9*).

Whether differences in antimicrobial drug use and race/ethnicity lead to disparities in antimicrobial drug resistance is unclear. We expect that disparities in use, regardless of their cause, will lead to disparities in the proportions of carried bacteria that are antimicrobial drug–resistant. Absolute rates of antimicrobial drug–resistant infections, however, might follow different patterns (*10*). For example, higher macrolide use among white persons might lead to macrolide resistance in a greater proportion of *Streptococcus pneumoniae* bacteria carried by whites, but if white persons have fewer *S. pneumoniae* infections, then they would incur a lower absolute rate of macrolide-resistant *S. pneumoniae* infections. Further studies comparing antimicrobial drug use, antimicrobial drug resistance, and disease prevalence by race/ethnicity will be critical for addressing this question and improving antimicrobial drug stewardship.

Technical Appendix Description of analytical methods and antimicrobial drug information.
