# Supplementary material for: Racial/Ethnic Disparities in Antimicrobial Drug Use, United States, 2014–2015
Source: Emerg Infect Dis. 2018 Nov;24(11):2126–8. doi: 10.3201/eid2411.180762 (PMC6199984; doi:10.3201/eid2411.180762)
Supplement: Technical Appendix — Description of analytical methods and antimicrobial drug information. [file 18-0762-Techapp-s1.pdf]

Article DOI: https://doi.org/10.3201/eid2411.180762

Racial/Ethnic Disparities in Antimicrobial Drug Use, United States, 2014–2015

Technical Appendix

We excluded persons with zero survey weight (variables PERWT14F and PERWT15F in the full-year consolidated data files). The categorical race variable was drawn from the RACETHX variable in the full-year consolidated data files, which encodes the 5 categories listed in the main text. The dichotomous white race variable was drawn from the RACEWX variable in the same file, which encodes 3 categories: only white race reported, white race and other races reported, and white race not reported. Antimicrobial drug fills were identified in the prescribed medicines file by matching the category identifications with any of the therapeutic class (TC) variables (e.g., TC1, TC1S1, TCS1_2) (Technical Appendix Table). CIs for the ratio X/Y between the fill rates X and Y for 2 different exposure groups were estimated by simulation. One million random deviates x_i were sampled from a normal distribution, with mean μ_X and standard deviation σ_X, where μ_X is the point estimate for X and σ_X is the standard error on X derived accounting for the complex survey design. One million deviates y_i were analogously sampled by using the point estimate and variance for Y. The 2.5%–97.5% interval of the distribution of the 1 million simulated ratios ($r_i = x_i/y_i$) was reported as the 95% CI for the ratio X/Y. The point estimate for the ratio is the mean of r_i. Code to reproduce the results is available at https://github.com/gradlab/abx-race.

Technical Appendix Table. Antimicrobial drugs included in study of racial/ethnic disparities in antimicrobial drug use, United States, 2014–2015

Multum Lexicon category ID no.	Antimicrobial drug description
002	Amebicide
008	Carbapenems
009	Cephalosporins
010	Leprostatic agents
011	Macrolides
012	Miscellaneous
013	Penicillins
014	Quinolones
015	Sulfonamides
016	Tetracyclines
017	Urinary anti-infective drugs
018	Aminoglycosides
240	Lincomycin derivatives

ID, identification.
